# High LRIG1 expression predicts lymph node metastasis in patients with uterine cervical cancer

**DOI:** 10.1002/2211-5463.70092

**Published:** 2025-07-23

**Authors:** Pernilla Israelsson, Lisa Lif, Husam Oda, Alexandra Lorenzzi de Melo, David Lindquist, Håkan Hedman

**Affiliations:** ^1^ Department of Diagnostics and Intervention, Oncology Umeå University Hospital Umeå Sweden; ^2^ Department of Medical Biosciences, Pathology Umeå University Hospital Umeå Sweden; ^3^ Department of Clinical Sciences, Professional Development Umeå University Hospital Umeå Sweden

**Keywords:** HPV, LRIG1, lymph node metastasis, p16, uterine cervical cancer

## Abstract

Fifteen percent of patients with preoperative stage IA2‐IB1 uterine cervical cancer are diagnosed with lymph node metastasis (LNM) following surgery. They must be treated with both surgery and radiotherapy, a combination associated with severe side effects. Since current diagnostic methods have limitations, biomarkers are urgently needed to improve staging. Leucine‐rich repeats and immunoglobulin‐like domains protein 1 (LRIG1) is a regulator of growth factor signaling and a prognostic factor in cervical cancer. This study investigates whether LRIG1 expression could predict LNM in cervical cancer. Sixty‐seven patients were included: 31 without LNM and 36 with LNM. Tumor blocks were retrieved, and clinical data were collected. Immunohistochemical analysis of LRIG1 expression was performed, and LRIG1 immunoreactivity was correlated with lymph node status and clinicopathological prognostic factors, such as human papillomavirus status and smoking status. High LRIG1 expression (> 25% positive cells) was significantly associated with an increased risk of LNM (odds ratio 9.49, 95% CI: 1.80–50.05, *P* = 0.008, adjusted for age, smoking status, and BMI), suggesting the potential of LRIG1 as a biomarker. Larger, multicenter studies are needed to validate our results.

## Graphical abstract

Lymph node status is crucial in determining treatment for women with early‐stage cervical cancer. We demonstrate that high LRIG1 protein expression in primary tumors can predict lymph node metastases. Our findings support further investigation of LRIG1 as a biomarker to improve staging accuracy and guide treatment decisions in cervical cancer patients.

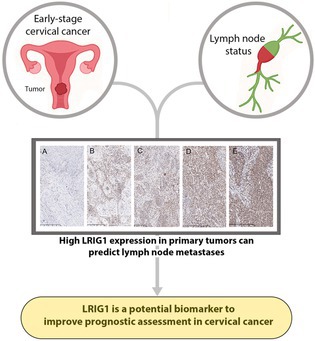

**Abbreviations**
BMI: body mass index
BMP: bone morphogenetic protein
CT: computed tomography
CTRT: chemoradiotherapy
EGFR: epidermal growth factor receptor
FFPE: formalin‐fixed and paraffin‐embedded
FIGO: fédération internationale de gynécologie et d'obstétrique
HPV: human papillomavirus
IHC: immunohistochemistry
LNM: lymph node metastasis
LRIG: leucine‐rich repeats and immunoglobulin‐like domains
MRI: magnetic resonance imaging
OR: odds ratio
PET: positron emission tomography

Approximately 660 000 new cases of uterine cervical cancer are reported every year. More than half of the patients will succumb to the disease, making it the fourth most common cause of cancer‐related death in women worldwide [1]. Almost all cases are induced by persistent human papillomavirus (HPV) infection [2]. The treatment for cervical cancer varies depending on the tumor stage, histological subtype, presence of recurrence risk factors, and comorbidities [3]. Currently, curative treatment options include surgery or radiotherapy. Studies examining the best treatment modality [4, 5, 6, 7, 8, 9] have failed to establish a clear consensus on the optimal treatment approach for each stage of the disease. However, the risk of severe side effects significantly increases when multiple treatment modalities are used, that is, surgery followed by radiotherapy [4, 5, 6]. Therefore, the current recommendation is to avoid the combination of surgery and radiotherapy. Currently, the Swedish recommendation for treating early‐stage cervical cancer is surgery [10], and this recommendation is based on a study showing no significant difference in survival between stage IB‐IIA patients receiving radical hysterectomy and radiotherapy [9]. For locally advanced stages, definitive chemoradiotherapy (CTRT) is recommended due to the increased likelihood of lymph node involvement to avoid treatment with more than one modality [10].

Lymph node spread has emerged as one of the most important prognostic factors, significantly impacting recurrence risk and survival outcome [7, 11, 12]. In patients with preoperative stage IB‐IIA disease, the 5‐year survival rate without lymph node involvement is 88–95%, whereas it is only 51–78% in patients for whom lymph node metastasis (LNM) is incidentally discovered following surgery [13]. Therefore, suspected lymph node spread is a clear indicator for recommending CTRT rather than surgery. In 2018, the *Fédération Internationale de Gynécologie et d'Obstétrique* (FIGO) staging system was revised, categorizing patients with lymph node spread as having at least stage IIIC disease, emphasizing the importance of lymph node involvement [14]. For correct staging and treatment planning to minimize overtreatment and the risk for severe side effects, accurate preoperative determination of LNM is essential. The current approach includes computed tomography (CT), magnetic resonance imaging (MRI), and positron emission tomography (PET) [10]. However, these imaging modalities have limitations in detecting lymph node involvement, with sensitivities of 40%, 48%, and 80%, respectively [15]. In fact, studies have shown that the false‐negative rate for PET is as high as 22% [16]. Hence, there is an urgent need for new and effective biomarkers that can reliably predict lymph node spread.

The leucine‐rich repeats and immunoglobulin‐like domains (LRIG) gene family encodes the three transmembrane proteins: LRIG1, LRIG2, and LRIG3 [17]. The LRIG proteins are expressed in all tissues that have been analyzed [18, 19, 20]. LRIG1 plays a role in the regulation of growth factor signaling and is a tumor suppressor in many cancer types. These effects are exerted through different mechanisms, including the enhancement of ubiquitylation and degradation of growth factor receptors from the ErbB family, including epidermal growth factor receptor (EGFR) [18, 21], and the promotion of bone morphogenetic protein (BMP) signaling [22]. Based on these findings, LRIG1 has been extensively studied as a potential prognostic marker in various malignancies. Hence, LRIG1 expression is associated with better survival in head and neck cancer [23], breast cancer [24], non‐small‐cell lung cancer [25], and cervical cancer [26, 27]. In prostate cancer, LRIG1 expression was associated with better survival in two American cohorts [28, 29] and with poor survival in a Swedish cohort where the patients were monitored by watchful waiting [28]. Since LRIG1 expression was a prognostic factor in cervical cancer, we wanted to investigate whether LRIG1 expression might also predict LNM in cervical cancer patients.

In this report, we show that LRIG1 expression in primary cervical cancer tumor tissue is predictive of lymph node spread.

## Materials and methods

### Patients

The study included women with uterine cervical cancer (ICD C53.9) who underwent a Wertheim–Meigs operation (radical hysterectomy and pelvic lymph node dissection) at Umeå University Hospital between 1997 and 2021. The patients were identified through patient records and selected according to lymph node status, with the required sample size for statistical power calculated using a 5% significance level. The expected effect size of LRIG1 expression was based on a previous study on ovarian cancer. To achieve statistical power of 80%, it was estimated that 39 patients in each group, including a LN‐positive and a LN‐negative group, would be required. Among 90 eligible patients, six were excluded because of other diagnoses (ICD C54.9), and 17 patients were excluded because of missing pathological anatomical diagnosis (PAD), leaving 36 patients in the LN‐positive group and 31 patients in the LN‐negative group. The study was conducted in accordance with the declaration of Helsinki and approved by the Regional Ethical Review Board at Umeå University (Dnr 2012‐229‐31M, 2013‐314‐32M, and 2022‐01127‐01). Informed verbal consent was obtained at the time of diagnosis, and samples were stored in the biobank for future research. No additional consent was required for this study, as approved by the Ethical Review Board. Formalin‐fixed and paraffin‐embedded (FFPE) tumor tissue samples were retrieved from the biobank at the Västerbotten County Council. Clinical data studied included age, body mass index (BMI), smoking habits, age at the time of diagnosis, histology, tumor grade (according to FIGO 2018) pre‐ and postoperatively, LNM status, postoperative TNM staging, primary treatment, time of recurrence, treatment at recurrence, and 5‐year disease‐free survival.

### Immunohistochemistry

Immunohistochemistry (IHC) was performed with the Ventana standard procedure in a Ventana Benchmark ULTRA (Ventana Medical Systems, Tucson, AZ, USA). Briefly, 4‐μm FFPE sections were stained following a protocol that included deparaffinization with CC2, followed by incubation with primary and secondary antibodies. For LRIG1, the primary LRIG1‐Vina antibody (product no. AS184165; AgriSera AB, Vännäs, Sweden) was used at a 1 μg·mL^−1^ concentration. The LRIG1‐Vina antibody was validated with LRIG1‐ablated HEK293T cells [30] as the negative control and HEK293T cells with a doxycycline‐inducible LRIG1 allele as the positive control (Fig. S1). For the HPV surrogate marker p16, an anti‐p16INK4a (E6H4) antibody (Ventana Medical Systems, Inc.) was used according to the manufacturer's protocol, with the incubation time extended from 12 to 24 min. Immunohistochemical slides were scanned using the Hamamatsu Nanozoomer S360 (Hamamatsu Photonics Norden AB, Kista, Sweden) with 40× optical magnification.

### Evaluation and classification of immunostaining

Immunohistochemical staining was evaluated and interpreted by a pathologist who subspecialized in gynecological oncology (HO) and was blinded to patient clinical data and outcomes. LRIG1 expression was quantified by determining the percentage of cells staining positive (absent, 1–25%, 26–50%, 51–75%, or 76–100% positive staining) and the staining intensity (none, low, intermediate, or high). Samples were scored as being positive for p16 expression if they showed strong and consistent expression in both the nucleus and cytoplasm in more than 70% of the cells [31]. In accordance with the journal's guidelines, we will provide our anonymized data for independent analysis by a selected team by the Editorial Team for the purposes of additional data analysis or for the reproducibility of this study in other centers if such is requested.

### Statistical analyses

Time to recurrence was defined as the time from the date of diagnosis to pathologically confirmed relapse of cervical cancer. If the exact date of any event was missing, the date was set to the first of the known month or year. Patient characteristics were analyzed using the independent samples *t*‐test to compare means or the chi‐squared test or Fisher's exact test to compare ordinal variables. Two‐sided *P* values are reported. Logistic regression was used to estimate the odds ratio (OR) for the associations among lymph node involvement, LRIG1 expression, and staining intensity. *P* values < 0.05 were considered to indicate statistically significant differences. All the statistical analyses were performed using SPSS 28 software (IBM SPSS Statistics 29, IBM, Armonk, NY, USA).

## Results

### Study population

Sixty‐seven patients with uterine cervical cancer who underwent Wertheim–Meigs surgery (radical hysterectomy and pelvic lymph node dissection) at Umeå University Hospital, Sweden, between 1997 and 2021 were included. The main patient characteristics are summarized in Table 1. The mean and median ages and BMI did not differ significantly between the LN‐positive and LN‐negative groups. Smoking habits differed between the groups, although not significantly: 10 (29%) patients in the LN‐positive group were smokers, and three (10%) in the LN‐negative group were smokers (*P* = 0.062). Patients were staged according to FIGO 1994, 2009, or 2018, depending on the year of diagnosis. All the patients were restaged to FIGO 2018 both pre‐ and postoperatively. Preoperative staging (Table 1) revealed, as expected, only early‐stage disease, indicating that all the included patients had undergone surgery. LN‐positive patients were postoperatively assigned stage IIIC disease. Forty‐one of the 67 patients (61%) were diagnosed with squamous cell carcinoma, 23 (34%) with adenocarcinoma, two (3%) with adenosquamous carcinoma, and one (1.5%) with large cell neuroendocrine carcinoma. Forty‐seven patients received adjuvant radiotherapy, with 28 patients receiving concomitant chemotherapy (Table 1). During the following 5 years of follow‐up, eight deaths were reported, one in the LN‐negative group (from cancer) and seven in the LN‐positive group (six from cancer, one from a massive pulmonary embolism postoperatively). Cervical cancer patients without LNM had a significantly lower recurrence rate (*P* = 0.009): Two (7%) patients in the LN‐negative group relapsed within a mean of 7.5 years, and 11 (33%) patients in the LN‐positive group relapsed within a mean of 2 years.

**Table 1:** Clinicopathological characteristics of the patients. BMI, body mass index; FIGO, Fédération Internationale de Gynécologie et d'Obstétrique; LN‐negative, Lymph node negative; LN‐positive, Lymph node positive; LRIG, Leucine‐rich repeats and immunoglobulin‐like domain; NK, not known; TNM, Tumor node metastasis.

Variable		Frequency (%)			*P* value
		LN‐positive	LN‐negative	All	
*N*		36	31	67	
Age (years)	Range	22–81	25–80	22–81	
	Mean	49	55	52	0.111^a^
BMI	Range	16.7–37.4	19.0–32.5	16.7–37.4	
	Mean	25	25	25	0.935^a^
	NK	3	8	11	
Smoking habit	Smoker	10 (29)	3 (10)	13 (20)	0.062^b^
	Nonsmoker	25 (71)	27 (90)	52 (80)	
	NK	1	1	2	
Histology	Squamous	25 (69)	16 (52)	41 (61)	0.310^b^
	Adenocarcinoma	10 (28)	13 (42)	23 (34)	
	Other	1 (3)	2 (6)	3 (5)	
TNM stage postoperative	T1b1		16 (57)	16 (25)	
	T1b2		10 (35)	10 (16)	
	T1b3		1 (4)	1 (2)	
	T2a1		1 (4)	1 (2)	
	T1b1N1	11 (31)		11 (17)	
	T1b2N1	15 (42)		15 (23)	
	T1b2N2	1 (3)		1 (2)	
	T1b3N1	7 (19)		7 (11)	
	T2bN1	2 (5)		2 (3)	
	NK		3	3	
FIGO 2018 stage preoperative	IBx	4 (11)	2 (7)	6 (9)	
	IB1	12 (33)	20 (65)	32 (48)	
	IB2	16 (45)	5 (16)	21 (31)	
	IB3	3 (8)	1 (3)	4 (6)	
	IIA1	1 (3)	3 (10)	4 (6)	
FIGO 2018 stage postoperative	IB1		16 (57)	16 (25)	
	IB2		10 (36)	10 (16)	
	IB3		1 (4)	1 (2)	
	IIA1		1 (4)	1 (2)	
	IIIC1	34 (94)		34 (53)	
	IIIC2	2 (6)		2 (3)	
	NK		3	3	
Primary treatment	Surgery	1 (3)	19 (62)	20 (30)	
	Surgery + radiotherapy	13 (36)	6 (19)	19 (28)	
	Surgery + chemoradiotherapy	22 (61)	6 (19)	28 (42)	
Recurrence	Yes	11 (33)	2 (7)	13 (21)	0.009^b^ ^,^ *
	No	22 (67)	28 (93)	50 (80)	
	NK	3	1	4	
Recurrence treatment	Surgery	1 (17)		1 (14)	
	Radiotherapy	2 (33)		2 (29)	
	Chemotherapy	3 (50)		3 (43)	
	Chemoradiotherapy		1 (100)	1 (14)	
	NK	5	1	6	
Time until recurrence (months)	Range	6–121	17–169	6–169	
	Mean	24	93	35	0.077^a^
p16 expression	Positive	33 (92)	30 (97)	63 (94)	0.379^b^
	Negative	3 (8)	1 (3)	4 (6)	
LRIG1 expression	Weak	7 (20)	10 (37)	17 (27)	0.011^b^ ^,^ *
	1–25%	10 (29)	14 (52)	24 (39)	
	26–50%	8 (23)	2 (7)	10 (16)	
	51–75%	9 (26)	0	9 (15)	
	76–100%	1 (3)	1 (4)	2 (3)	
	NK	1	4	5	
LRIG1 intensity	None	7 (20)	10 (37)	17 (27)	0.320^b^
	Weak	24 (69)	15 (56)	39 (63)	
	Intermediate	4 (11)	2 (7)	6 (10)	
	NK	1	4	5	

^a^ Independent samples *t* test (2‐sided *P* value).

^b^ Chi^2^ test.

* *P* < 0.05.

### LRIG1 and P16 expression, LRIG1 intensity, and association to lymph node status

**Fig. 1:**
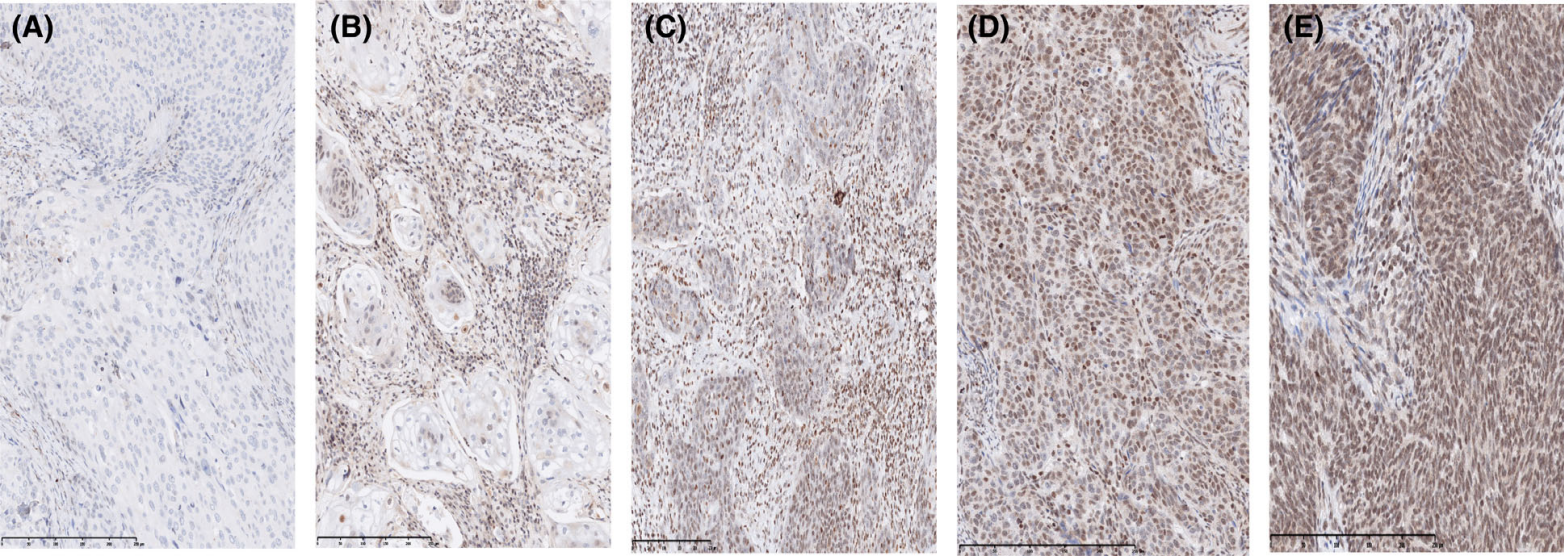
Micrographs showing examples of the different LRIG1 scores. Tissue sections from uterine cervical carcinoma were labeled with LRIG1 antibody (brown) and hematoxylin nuclear counterstain (blue). (A) No positive staining. (B) 1–25% positive tumor cells, weak staining intensity. (C) 26–50% positive tumor cells, weak staining intensity. (D) 51–75% positive tumor cells, weak staining intensity. (E) 76–100% positive tumor cells, intermediate staining intensity. Scale bars, 250 mm.

Staining for p16 was successful in all 67 tumors. LRIG1 staining failed in five tumors due to imperfect PAD (four without LNM and one with LNM). Among the 67 samples, 63 (94%) stained positive for p16, and four (6%) stained negative. There was no difference in p16 status between the LN‐positive and LN‐negative groups; only three (8%) of the LN‐positive samples and one (3%) of the LN‐negative samples were negative for p16 (*P* = 0.379, chi‐squared test). LRIG1 staining failed in five tumors due to imperfect PAD (four without LNM and one with LNM). LRIG1 immunoreactivity was predominantly nuclear (Fig. 1). Seventeen (27%) of the tumors showed no LRIG1 expression, 24 (39%) had 1–25% positive cells, 10 (16%) had 26–50%, nine (15%) had 51–75%, and two (3%) had 76–100% LRIG1‐positive cells. LRIG1 staining intensity was weak in 17 (27%), low in 39 (63%), and intermediate in six (10%) of the tumors, with no tumors showing strong staining intensity. Micrographs showing examples of different LRIG1 scores are shown in Fig. 1.

LRIG1 expression significantly differed between the LN‐positive and LN‐negative groups (*P* = 0.011, chi‐squared test) (Table 1). When the patients were dichotomized into the LRIG1‐high (> 25% LRIG1‐positive cells, *n* = 21) and LRIG1‐low (≤ 25% LRIG1‐positive cells, *n* = 41) groups according to median expression, a significant difference in LN status was observed (*P* < 0.001). Logistic regression analysis revealed that high LRIG1 expression was associated with an increased risk of LNM [OR 8.47, 95% confidence interval (CI): 2.15–33.37, *P* = 0.002]. When adjusted for age, smoking status, and BMI, the LRIG1‐high group still had a greater risk of LNM (adjusted OR 9.49, 95% CI: 1.80–50.05, *P* = 0.008) (Table 2). No significant difference in LRIG1 staining intensity was found between the LN‐positive patients and the LN‐negative patients according to the chi‐squared test (*P* = 0.320) (Table 1). The odds ratio for LRIG1 intensity (Table 2) showed no association with lymph node status (OR = 2.59, 95% CI: 0.65–10.25, *P* = 0.176). There was no correlation between p16 status and LRIG1 expression or intensity (Table S1).

**Table 2:** Association between LRIG1 intensity and expression and lymph node spread by logistic regression adjusted for smoking status, age, and BMI. LRIG, Leucine‐rich repeats and immunoglobulin‐like domain.

Variable		Odds ratio	*P* value	95% confidence interval
LRIG1 intensity	None	1		
	Low	2.59	0.176	0.653–10.252
	Intermediate	1.92	0.577	0.195–18.937
Lrig1 expression	0–25%	1		
	26–100%	9.487	0.008*	1.799–50.046

* *P* < 0.05.

## Discussion

We investigated whether LRIG1 expression could predict LNM in patients with cervical cancer. Indeed, high LRIG1 immunoreactivity in the primary tumor was significantly associated with LNM (adjusted OR 9.49, 95% CI: 1.80–50.05, *P* = 0.008). We also confirmed a significant difference in recurrence rates between LN‐positive and LN‐negative patients. There was a trend toward a greater proportion of smokers in the LN‐positive group. The clinicopathological characteristics of the cohort, such as the distribution of histopathological subtypes and age at diagnosis, were consistent with Swedish national statistics. We used p16 analyses as a surrogate marker for HPV status, as in the clinical routine [32, 33, 34]. As expected, almost all cases were p16‐positive, and there was no significant difference between the LN‐positive and LN‐negative groups (corresponding to different FIGO stages) (Table 1). Increased levels of p16 are caused by the inhibition of the transcription of cyclin‐dependent kinase inhibitor gene p16(INK4a) by viral oncogenes, and thus, using p16 as a surrogate marker does not discriminate between different HPV subtypes [33],

LNM is an important prognostic marker of cervical cancer [7, 10, 11, 12], which was confirmed by our data. Occasionally, patients are incorrectly identified as having early‐stage disease, and surgery is recommended. When LNM is discovered following surgery, adjuvant radiotherapy must be added, leading to multiple treatment‐related morbidities [6]. Therefore, a biomarker that can predict LNM is essential for minimizing overtreatment and reducing the risk of severe side effects.

Previous studies on LRIG1 expression in cervical cancer have focused on its potential for use as a prognostic marker [18, 26, 27]. Our results suggest that LRIG1 expression may also play a role in predicting LNM. We found that LRIG1 expression levels significantly differed between LN‐positive and LN‐negative patients, with high LRIG1 expression being associated with LNM. Previous studies on LRIG1 expression in cervical cancer reported that high LRIG1 expression was a positive prognostic factor in early‐stage squamous cell carcinoma [26] and adenocarcinoma [27]. While these findings may seem contradictory to our current results, it is essential to note that the designs and aims of the studies were entirely different. Specifically, whereas LRIG1 was evaluated for use as a prognostic marker in previous studies, here we investigated whether LRIG1 could predict LNM. Interestingly, similar apparently contradictory findings were reported in non‐small‐cell lung cancer, where high LRIG1 expression was associated with both a good prognosis [25] and mediastinal LNM [35]. Additionally, the subcellular localization of LRIG1 might be important for its biological function and prognostic implications. In the cervical carcinomas analyzed here, LRIG1 immunoreactivity was mainly nuclear. A nuclear LRIG1 staining pattern has been observed in several cancer types, for example, in basal cell carcinoma [18], non‐small‐cell lung cancer [25], and head and neck cancer [23]. Furthermore, LRIG1 subcellular localization might change according to the stage of the disease; for example, LRIG1 localization shifts from predominantly nuclear in normal skin to cytoplasmic in psoriatic lesions [36]. Hence, the prognostic value of LRIG1 expression appears to depend on the biological and cellular context and stage of the disease. Accordingly, in advanced stages of uterine cervical cancer, there was no correlation between LRIG expression and survival [26].

Smoking is a well‐known risk factor for both the development and progression of cervical cancer [37]. In our study, we observed a trend suggesting a greater proportion of smokers among the LN‐positive patients than among the LN‐negative patients. Although this trend was not statistically significant (*P* = 0.062), it suggests that smoking may be a contributing factor to the development of LNM. Previous studies have identified tumor‐specific risk factors for LNM, such as tumor size > 2 cm, lymphovascular invasion, deep stromal invasion, and parametrial involvement [38, 39]. To our knowledge, this is the first study to suggest that smoking could also be a risk factor for LNM.

The current study has several strengths, including the use of a well‐defined patient cohort and thorough collection and analysis of clinical data, such as tumor histology, staging, and treatment outcomes. These strengths provide a solid foundation for interpreting the findings. The representativeness of our dataset is supported by the similarity of the histopathological distribution and median age at diagnosis to national statistics for patients with uterine cervical cancer in Sweden [10, 40]. Limitations of the study include the fact that this was a retrospective study, making randomization impossible. The relatively small cohort size may limit the generalizability of the findings, especially for subgroup analyses. Furthermore, data from a single institution could introduce selection bias, which might affect the applicability of the results to broader populations.

Our results indicate that the use of LRIG1 expression as a potential biomarker for lymph node involvement in patients with cervical cancer should be further explored. The current study used surgically removed tumor tissues, but the anatomical position of cervical cancer offers a unique opportunity for preoperative diagnosis. Immunohistochemical staining of a cervical cancer biopsy sample could be used preoperatively to assess LRIG1 expression and guide treatment decisions. The integration of immunohistochemical biomarkers into clinical practice is complex and requires the fulfillment of specific criteria. Most importantly, the biomarker assay must be reproducible, and the information it provides must be clinically meaningful. In the present study, a relatively small cohort was analyzed using a polyclonal antibody against LRIG1. To enhance assay reproducibility, the use of a standardized detection reagent, such as a monoclonal antibody, would be preferable. Future efforts will determine whether a suitable monoclonal antibody targeting LRIG1 can be developed. Likewise, future retrospective and prospective studies in independent, larger cohorts will be necessary to assess whether LRIG1, either alone or in combination with other biomarkers, can reliably predict lymph node metastasis in early‐stage cervical carcinoma in a clinically meaningful way. A method for measuring LRIG1 levels in plasma was recently developed [41]. Studying LRIG1 blood levels in cervical cancer patients could provide a less invasive alternative to biopsy‐based testing that should be further evaluated for use as a biomarker. Lifestyle factors such as smoking have not been extensively studied in relation to LNM risk. Further research is needed to explore the potential biological mechanisms linking smoking to lymphatic spread.

## Conclusions

For the first time, we demonstrated that high LRIG1 immunoreactivity predicts LNM in cervical cancer patients (adjusted OR 9.49, 95% CI: 1.80–50.05, *P* = 0.008). To confirm these findings and potentially establish LRIG1 as a clinically relevant biomarker, future studies should focus on larger, multicenter cohorts to increase the statistical power and generalizability.

## Conflict of interest

The authors declare no conflict of interest.

## Author contributions

PI: conceptualization, methodology, software, formal analysis, investigation, data curation, writing—original draft, writing—review and editing, visualization, funding acquisition. LL: software, formal analysis, investigation, data curation, writing—original draft, visualization. HO: investigation, data curation, visualization, writing—review and editing. AL‐M: investigation, data curation, visualization, writing—review and editing. DL: conceptualization, writing—review and editing, supervision, project administration, funding acquisition. HH: conceptualization, methodology, resources, writing—review and editing, supervision, project administration, funding acquisition.

## Supporting information


**Fig. S1.** Validation of the Vina LRIG1 antibody.

**Table S1.** LRIG1 expression and staining intensity, and p16 status.

## Data Availability

The data that support the findings of this study are available upon request from the corresponding author. These data are not publicly available due to privacy or ethical restrictions.
