# Supplementary figures and images for: High LRIG1 expression predicts lymph node metastasis in patients with uterine cervical cancer
Source: FEBS Open Bio. 2025 Jul 23;15(12):2045–53. doi: 10.1002/2211-5463.70092 (PMC12667210; doi:10.1002/2211-5463.70092)

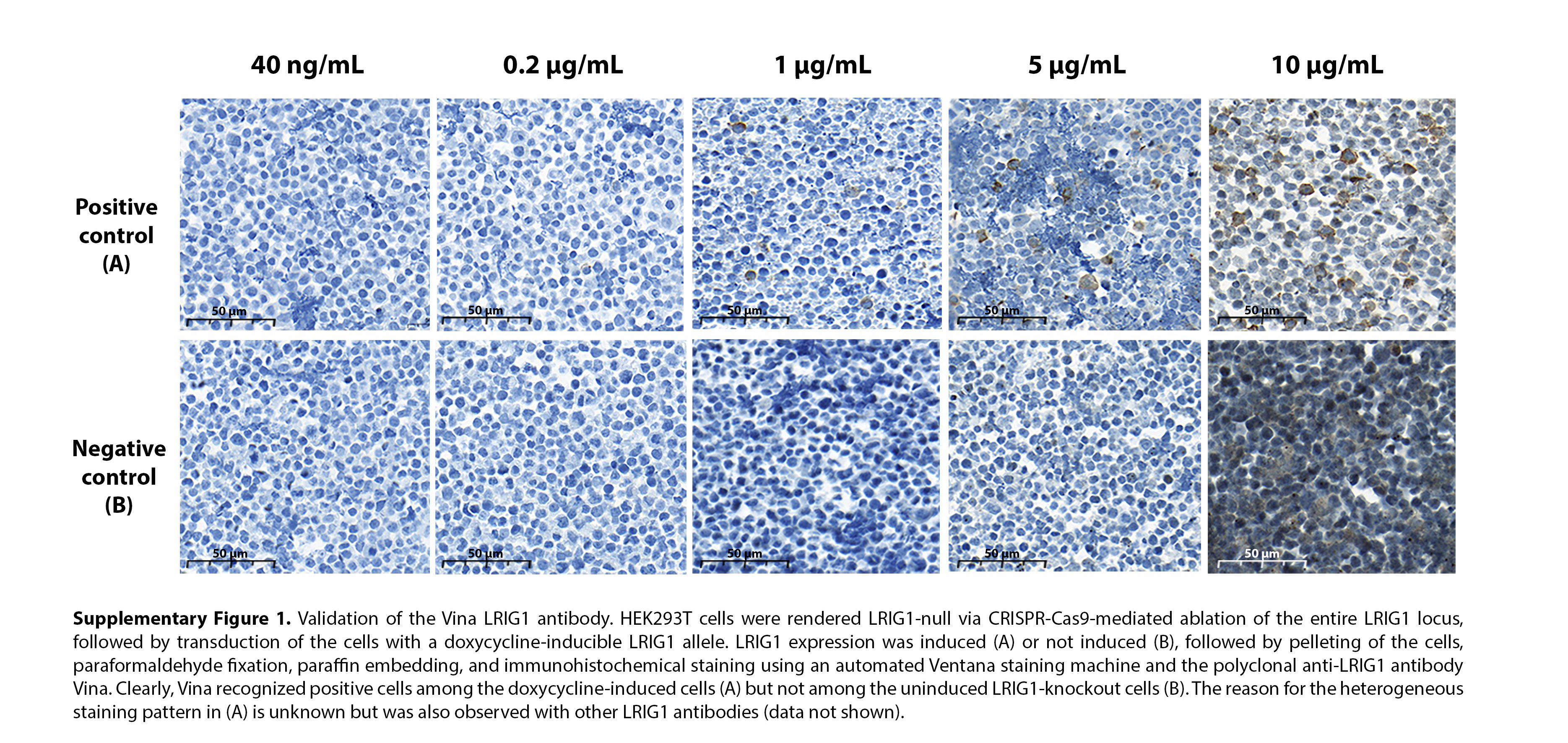

Supplement: Supplementary file 1 — Fig. S1. Validation of the Vina LRIG1 antibody. [file FEB4-15-2045-s002.tiff]
