# Supplementary material for: High LRIG1 expression predicts lymph node metastasis in patients with uterine cervical cancer
Source: FEBS Open Bio. 2025 Jul 23;15(12):2045–53. doi: 10.1002/2211-5463.70092 (PMC12667210; doi:10.1002/2211-5463.70092)
Supplement: Supplementary file 2 — Table S1. LRIG1 expression and staining intensity, and p16 status. [file FEB4-15-2045-s001.docx]

**Supplementary table 1.** LRIG1 expression and staining intensity, and p16 status.

| **Variable** | | **Frequency (%)** | | | |
| --- | --- | --- | --- | --- | --- |
| *N* | | P16 negative 3 | P16 positive 59 | All  62 | p value |
| LRIG1 expression | 0 | 0 (0) | 17 (29) |  | 0.227^a^ |
|  | 1-25% | 1 (33) | 23 (39) |  |  |
|  | 26-50% | 2 (67) | 8 (14) |  |  |
|  | 51-75% | 0 | 9 (15) |  |  |
|  | 76-100% | 0 | 2 (3) |  |  |
| LRIG1 intensity | None | 0 | 17 (29) |  | 0.285^a^ |
|  | Weak | 2 (67) | 37 (63) |  |  |
|  | Intermediate | 1 (33) | 5 (8) |  |  |

LRIG, Leucine-rich repeats and immunoglobulin-like domain
^a^Fisher’s exact test
* = *P* <0.05
